# Development of a droplet digital PCR method for the detection of *Ureaplasma urealyticum*

**DOI:** 10.1016/j.plabm.2024.e00443

**Published:** 2024-12-14

**Authors:** Yong-Zhuo Zhou, Yun-Hu Zhao, Yan-Lan Chen, Hua Luo, Yu-lin Zhou, Bing Gu, Wei-Zhen Fang, Chao-Hui Duan, Xu-Guang Guo

**Affiliations:** a Laboratory of Clinical, The Sun Yat-sen Memorial Hospital, Sun Yat-sen University, Guangzhou, 510120, China; b Department of Clinical Laboratory Medicine, Guangdong Provincial Key Laboratory of Major Obstetric Diseases, Guangdong Provincial Clinical Research Center for Obstetrics and Gynecology, The Third Affiliated Hospital of Guangzhou Medical University, Guangzhou, 510150, China; c Department of Clinical Medicine, The Third Clinical School of Guangzhou Medical University, Guangzhou, 511436, China; d Guangzhou Key Laboratory for Clinical Rapid Diagnosis and Early Warning of Infectious Diseases, King Med School of Laboratory Medicine, Guangzhou Medical University, Guangzhou, 510000, China; e Guangzhou Xiaoqi Biotechnology Co., LTD, Guangzhou, 510699, China; f Department of Laboratory Medicine, Guangdong Provincial People's Hospital (Guangdong Academy of Medical Sciences), Southern Medical University, Guangzhou, 510080, China

**Keywords:** Droplet digital PCR, Ureaplasma urealyticum, Quantitative testing

## Abstract

**Background:**

Human infection with *Ureaplasma urealyticum(UU)* is mainly manifested as non-gonococcal urethritis, where it can lead to cervicitis, premature rupture of membranes and abortion in women, as well as infertility in males, which becomes a major problem in clinical diagnosis and treatment. At present, real-time fluorescence quantitative PCR and culture are the two main methods for detecting UU. The real-time fluorescence quantitative PCR method is cumbersome and cannot accomplish absolute quantification on nucleic acids, while the cultivation method has limitations such as low sensitivity and being time-consuming. The aim of this study is to establish a more rapid and accurate droplet digital PCR(ddPCR) method for the detection of UU.

**Methods:**

Primers were designed for the ParC gene of UU. Nucleic acids from a standard strain of UU were extracted. Specificity, sensitivity, and repeatability detection was performed using ddPCR, and the detection performance of ddPCR was evaluated.

**Results:**

The detection process could be completed in 92 min. It has a high sensitivity of up to 3.8 pg/μL. With a high specificity, no positive microdrop were detected in eight negative control pathogens in this experiment. In addition, ddPCR detection of UU has good repeatability, and the calculated CV is 2.1 %.

**Conclusion:**

Our data indicated that ddPCR detection technology has the characteristics of absolute quantification, high stability, high specificity and high sensitivity of UU. It can promote the accurate detection of UU, providing a more scientific basis for clinical diagnosis and treatment.

## 1 Introduction

UU is susceptible to infection in both men and women. It has an incubation period of 1–3 weeks. Some patients do not develop obvious symptoms after infection and are not easily detected, which can easily lead to missed diagnosis and misdiagnosis [1]. Typical acute symptoms include urgency, frequency, and pain during urination. In men, infection can be superimposed with prostate infection, while in women, infection can lead to salpingitis, endometritis, pelvic inflammatory disease etc, besides being an important factor leading to infertility [2,3]. Domestic and foreign research data shows that the positive rate of UU in cervical mucus or semen of infertile couples is as high as 50 % [4]. In addition, UU infection is also an important factor leading to abortion and ectopic pregnancy, which warrants attention from a medical and health perspective [5,6]. At present, pathogen culture and real-time fluorescence quantitative PCR methods are mainly used for the detection of UU in clinical laboratorie [7]. However, the detection of pathogen culture is time-consuming and has a low sensitivity. The application of real-time fluorescence quantitative PCR method is becoming increasingly widespread, but the analysis procedures are cumbersome and cannot be absolutely quantified. Therefore, it is necessary to establish a simple and accurate method for the detection of UU.

Digital PCR is a novel technology that has been developed in recent years. DdPCR is a method of PCR amplification based on a single molecule template, and the whole reaction process does not require a standard curve to obtain the absolute copy number of the target gene in the reaction system [8,9]. DdPCR technology has been widely used in detecting a variety of bacteria and viruses, which has been useful for pathogen identification in clinics [[10], [11], [12], [13]]. In addition, relevant studies have shown that ddPCR has also been used in tumor typing and prenatal diagnosis [[14], [15], [16]].

Thus far, detection methods for *UU* have primarily relied on commercial qPCR. Compared with commercial qPCR methods, ddPCR can perform absolute quantification and is more accurate, which has important significance in treatment monitoring. In our study, We had evaluated the ddPCR for the detection of *UU.* we demonstrated that *UU* can be accurately detected by ddPCR.This method could be beneficial to the clinical diagnosis and treatment of *UU* infections.

## 2 Materials and methods

### 2.1 Bacterial strains

The standard strain of *UU* (ATCC33698)was obtained from the Shanghai Rendu Biotechnology Co., LTD. *Mycoplasma genitalium, Mycoplasma hominis, Chlamydia trachomatis, Enterococcus faecalis, Enterococcus faecium, Staphylococcus aureus, Ureaplasma parvum and Proteus mirabilis* were isolated and stored from the laboratory of clinical of Sun Yat-sen Memorial Hospital, Sun Yat-sen University. Mycoplasma genitalium, Mycoplasma hominis, and *Chlamydia trachomatis* were correctly identified in the PCR lab by SAT technology. Other bacteria are correctly identified in the microbiology lab by mass spectrometry.

### 2.2 Primer design and synthesis

Six sets of primers were designed based on the ParC gene (serial number EU279402.1) in NCBI(https://www.ncbi.nlm.nih.gov/) and synthesized by Shanghai Bioengineering Co., Ltd [17]. The primer sequence of this study are shown in Table 1.

**Table 1:** ParC primer used for the detection of UU.

Primers	Sequence (5’ →3′)	Length (bp)
ParC-1 upstream	ATCAGCACGTACAGTTGGGG	20
ParC-1 downstream	GCATCGCAGCAGCGTTATC	19
ParC-2 upstream	AATCAGCACGTACAGTTGGGG	21
ParC-2 downstream	TCTGTATAACGCATCGCAGC	20
ParC-3 upstream	AAATCAGCACGTACAGTTGGG	21
ParC-3 downstream	TTCTGTATAACGCATCGCAGC	21
ParC-4 upstream	CAGCACGTACAGTTGGGGAAG	21
ParC-4 downstream	CAGCAGCGTTATCACCATCAA	21
ParC-5 upstream	ATCAGCACGTACAGTTGGGGA	21
ParC-5 downstream	TTTCTGTATAACGCATCGCAGC	22
ParC-6 upstream	GCACGTACAGTTGGGGAAGTAA	22
ParC-6 downstream	CATCGCAGCAGCGTTATCA	19

### 2.3 Primer screening analysis

Six sets of primers were designed, the DNA of the ATCC33698 strain was served as the template, and Real time fluorescent quantitative PCR was performed. The reaction procedure was set to 50 °C 120s, 95 °C 150s for one cycle, and 94 °C 35s and 60 °C 35s for 40 cycles. The melting curves procedure was set to 95 °C 15s, 60 °C 60s, 95 °C 15s, and 60 °C for 15s. Finally, compare with the amplification efficiency of six sets of primers, the primers with high amplification efficiency and no primer dimer were selected for the subsequent assay.

### 2.4 Droplet digital PCR reaction

According to the reagent instructions, each test was prepared with a 25 μL reaction system, including 12.5 μL PCR reagent, 1 μL forward and reverse primers, 6 μL DNA template, and 4.5 μL ultrapure water.14 μ L mixture and 15 μ L oil was added into the droplet generator, until completed. The reaction procedure was the same as that of qPCR amplification except for part of the melting curve. Chip digital PCR was used to complete the amplification reaction in the TC1 digital PCR instrument. Finally, the fluorescence signal in each plate was analyzed by the biochip reader CS7 [18].

### 2.5 The sensitivity of ddPCR reaction

The Thermo Scientific Nanodrop 2000 microspectrophotometer was adjusted with TE buffer and the nucleic acid concentration of UU was detected. 10 μL of nucleic acid stock solution was diluted 10-fold with ultrapure water to obtain five template concentrations. The ddPCR reaction was performed with five concentrations to determine the sensitivity of ddPCR for detecting UU.

### 2.6 Specificity and repeatability of ddPCR for UU

Under the same amplification procedure, UU standard strain and other negative control bacterial nucleic acids were amplified by ddPCR. Evaluate the specificity of primers. At the same time, the UU standard strain and negative strains were detected three times with ddPCR under the same conditions. The fluorescence signal was analyzed to evaluate the reliability of ddPCR for the detection of UU.

## 3 Results

### 3.1 Primer screening analysis

**Fig. 1:**
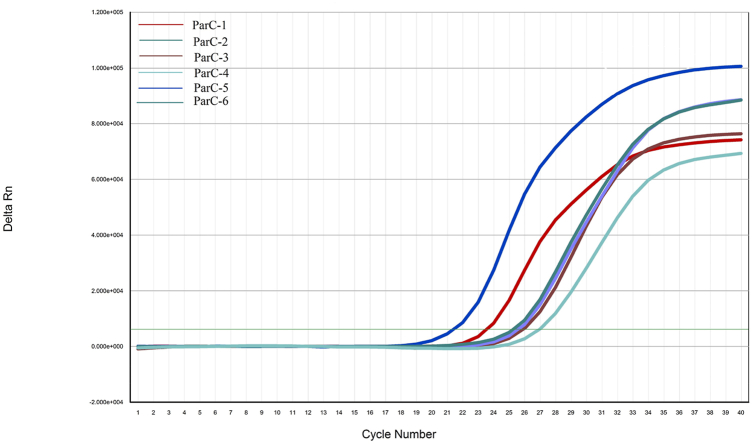
The primer of the qPCR screening experiment.

**Fig. 2:**
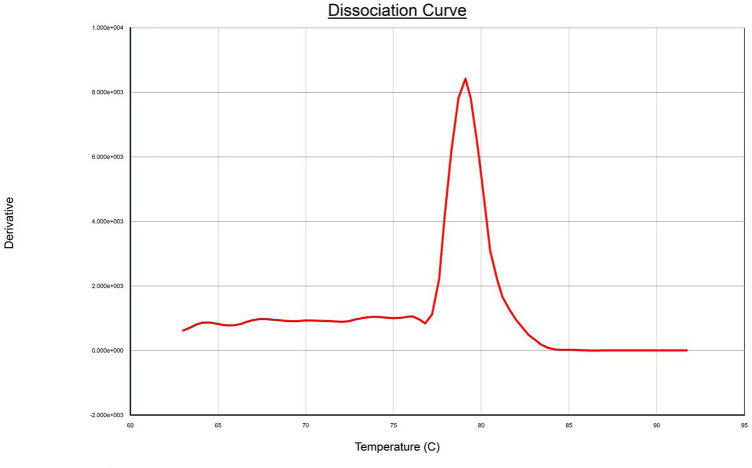
The melting curve of primer ParC-5.

In this study, primer screening tests were performed to UU standard strain ATCC33698 by qPCR. and the results showed that primer ParC-5 had the highest amplification efficiency, and its Ct value was 21 (Fig. 1). The qPCR melting curve showed a single peak, indicating the absence of nonspecific amplification and primer dimer of the reaction (Fig. 2). Although the melting curve of the other five sets of primers had a single peak, their amplification efficiency was lower than that of ParC-5 (data not shown). Therefore, ParC-5 was chosen for follow-up tests.

### 3.2 Specificity of ddPCR for UU

**Fig. 3:**
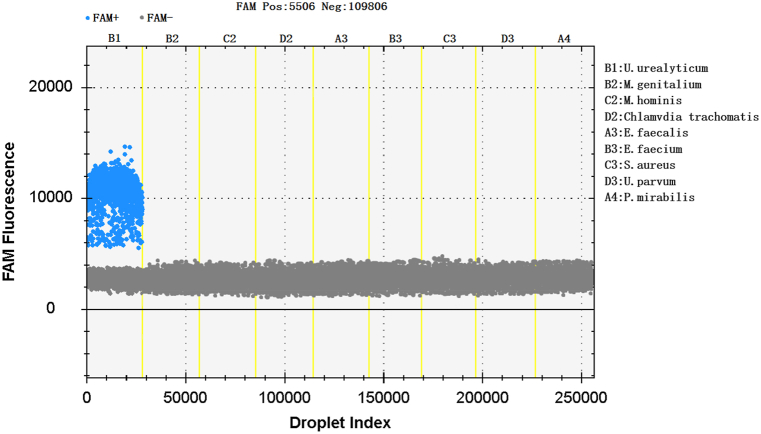
Specificity of ddPCR for the amplification of UU.

The genomic DNA of UU standard strain ATCC33698 and eight negative control bacteria were detected by ddPCR at the same time. Positive droplets could only be detected for UU, while positive droplets could not be detected for eight negative control bacteria. The results showed that ddPCR detection of ParC-5 gene of UU had good specificity and did not display cross-reactivity with non-target genes (Fig. 3). The horizontal coordinate represented the droplet index, and the vertical coordinate represented the fluorescence intensity detected by the FAM channel. Negative and positive droplets are shown in gray and blue.

### 3.3 Sensitivity of ddPCR for UU

**Fig. 4:**
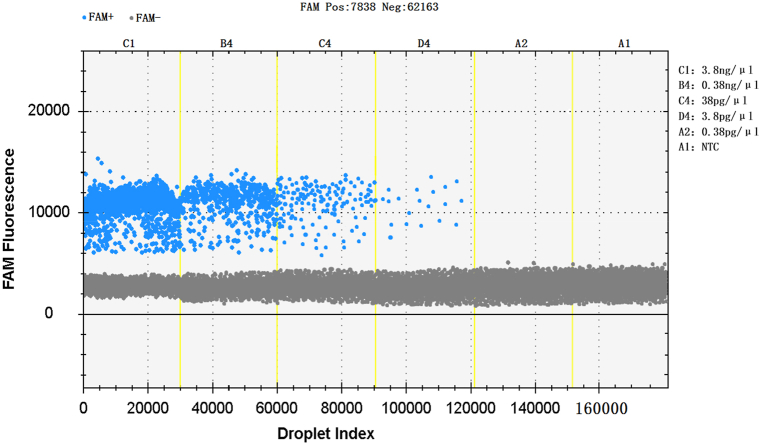
Sensitivity of ddPCR for the amplification of UU.

The nucleic acid concentration of the extracted UU standard strain ATCC33698 was measured by the Thermo Scientific Nanodrop 2000 microspectrophotometer, and the concentration was 3.8 ng/μL.After four 10-fold dilutions, the concentrations were 0.38 ng/μL, 38 pg/μL, 3.8 pg/μL and 0.38 pg/μL, respectively. The results of this experiment showed that the sensitivity reached 3.8 pg/μL (Fig. 4).

### 3.4 Reproducibility of ddPCR for UU

**Fig. 5:**
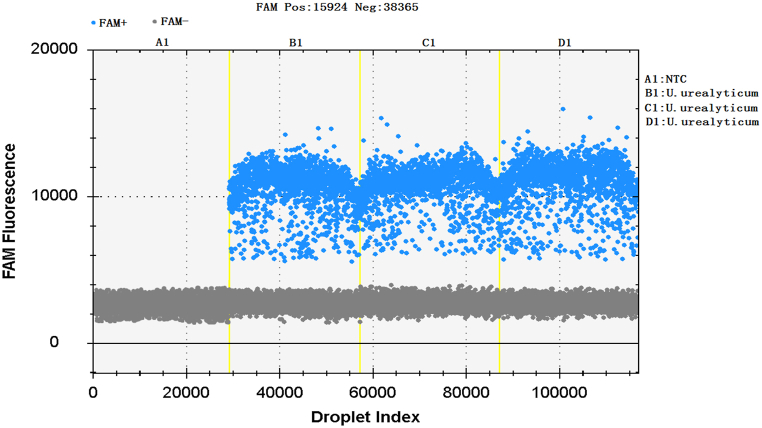
Reproducibility of ddPCR for the amplification of UU.

The results of ddPCR detection of UU ATCC33698 were 192, 194 and 200copies/μL, respectively, and the number of positive microdrops was similar. The CV was 2.1 % after calculation, which had good reproducibility. (Fig. 5).

## 4 Discussion

UU is a prokaryotic microorganism that is difficult to stain with Gram staining and Giemsa staining. Most patients have no obvious symptoms after infection, which can easily lead to missed diagnosis. Some patients present with urethritis, perineal swelling and pain, groin discomfort, and lumbar pain. However, the above symptoms are not specific. In addition, infection in pregnant women can lead to adverse consequences such as miscarriage and premature birth [19]. At present, bacterial culture is time-consuming with limited sensitivity for identifying UU infection. Real time fluorescence quantitative PCR detection cannot achieve absolute quantification of the target gene, and the analysis of result is relatively cumbersome. As a novel technology, ddPCR can perform absolute nucleic acid quantification without relying on standard curves, and is hindered by fewer influencing factors. Compared with bacterial culture and real-time fluorescence quantitative PCR, it has better diagnostic performance.

In our study, the fluorescent dye SYBRGreen which binds to dsDNA was used to monitor UU. If there was non-specific amplification or primer dimerization, false-positive results may occur or low amplification efficiency. Therefore, the melting curve was analyzed and no double-peak was found, indicating the absence of non-specific amplification or primer dimerization. We screened a set of primers that could efficiently amplify ParC genes. CT values of >35 suggest the failure of DNA extraction or amplification. We found that ddPCR amplification was highly efficient and the entire detection process could be completed in 92 min, including the time consumed for nucleic acid extraction. Compared to qPCR, the detection time is shortened. The high sensitivity of ddPCR for the detection of UU was satisfactory and could achieve accurate detection to as low as 3.8 pg/μL.Amplification was not observed in non-UU strains, which indicated the high specificity of ddPCR primers. The reproducibility test was very good, with a CV of 2.1 %. Given the above advantages, this method may be used to determine the expression of target genes and analyze their changes [20].

However, limitations are also inevitable. Firstly, aerosol pollution and primer dimer formation could significantly interfere with experimental results. Therefore, primer selection and experimental operation demand higher requirements. Secondly, it was reported that the ureaseB gene was targeted with LAMP to identify UU [21]. The choice of gene for the enhanced identification of UU could be further tested and compared. Thirdly, We have not yet completed clinical sample validation trials. More samples and different sample types need to be collected for further validation in the future.

Our study shows that the detection of UU by ddPCR has the characteristics of high specificity, sensitivity and reproducibility, and allow absolute quantification. However, a large number of clinical samples and different sample types would need to be collected for further validation, confirm its value in diagnostic and support that it is one of the clinical methods for the detection of UU.

## CRediT authorship contribution statement

**Yong-Zhuo Zhou:** Conceptualization, Data curation, Formal analysis, Resources, Writing – original draft, Writing – review & editing, Investigation, Project administration. **Yun-Hu Zhao:** Software, Supervision, Validation. **Yan-Lan Chen:** Formal analysis, Investigation, Software. **Hua Luo:** Project administration, Software, Visualization. **Yu-lin Zhou:** Investigation, Methodology, Project administration. **Bing Gu:** Data curation, Formal analysis. **Wei-Zhen Fang:** Resources, Supervision, Validation. **Chao-Hui Duan:** Resources, Validation, Visualization. **Xu-Guang Guo:** Conceptualization, Data curation, Resources, Project administration.

## Ethics approval and consent to participate

The study was approved by the ethics committees of the Sun Yat-sen Memorial Hospital, Sun Yat-sen University.

## Funding sources

Guangdong Basic and Applied Basic Research Foundation (2024A1515011037).

## Declaration of competing interest

The authors declare that they have no known competing financial interests or personal relationships that could have appeared to influence the work reported in paper.

## Data Availability

All data generated or analyzed during this study are included in this published article.

## Abbreviations

UU: Ureaplasma urealyticum; ddPCR: droplet digital PCR; CV:Coefficient of Variation.
